# Mesenchymal stem cells exert anti-proliferative effect on lipopolysaccharide-stimulated BV2 microglia by reducing tumour necrosis factor-α levels

**DOI:** 10.1186/s12974-014-0149-8

**Published:** 2014-09-03

**Authors:** Shinsmon Jose, Shi Wei Tan, Yin Yin Ooi, Rajesh Ramasamy, Sharmili Vidyadaran

**Affiliations:** Neuroinflammation Group, Immunology Laboratory, Department of Pathology, Faculty of Medicine and Health Sciences, Universiti Putra Malaysia, 43400 Serdang, Selangor Malaysia; Genetic Medicine Research Centre, Faculty of Medicine and Health Sciences, Universiti Putra Malaysia, 43400 Serdang, Selangor Malaysia; Current address: Tissue Engineering Centre, Universiti Kebangsaan Malaysia Medical Centre, 56000 Kuala Lumpur, Malaysia; Department of Anatomy, Yong Loo Lin School of Medicine, NUS, MD10, 4 Medical Drive, Singapore, 117597 Singapore; Stem Cells & Immunity Group, Immunology Laboratory, Department of Pathology, Faculty of Medicine and Health Sciences, Universiti Putra Malaysia, 43400 Serdang, Selangor Malaysia

**Keywords:** Microglia, Mesenchymal stem cells, Nitric oxide, Cell cycle, Interleukin-6, Tumour necrosis factor-α

## Abstract

**Background:**

Progression of neurodegenerative diseases occurs when microglia, upon persistent activation, perpetuate a cycle of damage in the central nervous system. Use of mesenchymal stem cells (MSC) has been suggested as an approach to manage microglia activation based on their immunomodulatory functions. In the present study, we describe the mechanism through which bone marrow-derived MSC modulate the proliferative responses of lipopolysaccharide-stimulated BV2 microglia.

**Methods:**

BV2 microglia were cultured with MSC and stimulated with 1 μg/ml lipopolysaccharide. Using an inducible nitric oxide synthase inhibitor, tritiated thymidine (^3^H-TdR) incorporation assay was performed to determine the role of nitric oxide in the anti-proliferative effect of MSC. We also studied apoptosis and the cell cycle of both cell types using flow cytometry and explored their cytokine profile using protein and cytometric arrays. Moreover, the role of IL-6 and TNF-α in immunomodulation was deduced using specific blocking antibodies and recombinant proteins.

**Results:**

MSC reduces microglia proliferation upon lipopolysaccharide stimulation by 21 to 28% and modulates the levels of nitric oxide, IL-6 and TNF-α. The role of nitric oxide in conferring the anti-proliferative effect of MSC was ruled out. Furthermore, we found that MSC exert their anti-proliferative effect by restoring the percentage of BV2 cells at S and G2/M phase to levels similar to unstimulated cells. MSC undergo a G0/G1 arrest while exerting this effect. We have also identified that MSC-mediated modulation of microglia is independent of IL-6, whilst reduction of TNF-α in co-culture is critical for inhibition of microglia proliferation.

**Conclusions:**

Our study demonstrates that MSC inhibit microglia proliferation independent of nitric oxide and IL-6, although reduction of TNF-α is critical for this effect. The inhibition of proliferation is through cell cycle modulation. These findings shed light on the mechanisms of microglial immunomodulation by MSC.

**Electronic supplementary material:**

The online version of this article (doi:10.1186/s12974-014-0149-8) contains supplementary material, which is available to authorized users.

## Background

Mesenchymal stem cells (MSC) are a group of clonogenic, non-haematopoietic, plastic adherent multi-potent stromal cells that possess potential to differentiate into cells of mesodermal lineage [1,2]. MSC have been shown to ameliorate disease in numerous clinical trials and animal models. Approximately 400 clinical trials using MSC are currently underway according to the clinical trials registry of the US National Institute of Health (http://clinicaltrials.gov). It is noteworthy that a good proportion of these studies are exploring the immunomodulatory properties of MSC. This aspect of MSC is widely explored in managing graft-versus-host disease [3] and in a variety of experimental disease models, including autoimmune encephalomyelitis [4], stroke [5], amyotrophic lateral sclerosis [6], spinal cord injury [7], diabetes [8] and myocardial infarction [9]. These advances at the therapeutic level are a result of detailed descriptions on the potential of MSC to modulate a range of immune cells including T cells, B cells, dendritic cells, monocytes and macrophages [10-15].

Microglia are resident macrophages of the central nervous system (CNS), derived from primitive myeloid progenitors that migrate from the embryonic yolk sac to the brain rudiment [16]. In adults, microglia survey the entire brain every couple of hours through concerted movements [17] for damaged neurons, endogenous disease proteins, and infection [18]. Microglia assume functions beyond being immune sentinels by maintaining a healthy CNS environment through synaptic pruning [19-21] and secretion of neurotrophic factors [22,23]. In response to disturbances in CNS homeostasis microglia undergo morphological, phenotypic and functional changes. These changes include an increase in numbers through proliferation, a shift from ramified to amoeboid morphology, secretion of inflammatory mediators such as cytokines, chemokines and reactive oxygen and nitrogen species, and an increase in phagocytic activity. Experiments have shown that these changes which microglia undergo upon activation can cause deleterious effects within the CNS microenvironment and play a key role in pathogenesis of neurodegenerative disease [24-27].

Modulating microglia responses is being considered as an effective approach to manage progression of neurodegenerative diseases. Recently, with the rise in reports on immunomodulatory prospects of MSC, we and others have explored and described the potential of MSC to dampen inflammatory responses of microglia [28-32]. However, the mechanisms underlying these effects are poorly understood. The present study determined the mechanisms through which MSC confer an anti-proliferative effect on microglia by examining apoptosis and cell cycle. We also determined the role of nitric oxide, IL-6 and TNF-α in conferring the modulatory effects of MSC. It was also demonstrated that MSC experience growth arrest in co-culture while modulating microglial responses.

## Materials and methods

### Mouse bone marrow mesenchymal stem cell culture

MSC previously isolated from ICR mouse bone marrow were obtained from the culture collection of the Immunology Laboratory, Faculty of Medicine and Health Sciences, Universiti Putra Malaysia. Frozen vials stored in liquid nitrogen were thawed at 37°C and cultured in MSC complete medium (high glucose DMEM (GIBCO Invitrogen, CA, USA), supplemented with 15% (v/v) MesenCult® Mesenchymal Stem Cell Stimulatory Supplements (Mouse) (Stemcell™ Technologies, Canada), 1% penicillin and streptomycin (iDNA), 250 μg/ml fungizone (GIBCO Invitrogen, CA, USA), 2.0 mM GlutaMaX and 1.5 g sodium bicarbonate) at 37°C, 5% CO_2_ in a humidified incubator. Cells were routinely sub-cultured using 0.25% Trypsin-EDTA (GIBCO Invitrogen, CA, USA) before reaching 90% confluency and immunophenotyped using a panel of markers comprising CD106, CD45, CD44, CD11b, Sca-1, MHC-I and MHC-II (all from Becton Dickinson, BD, San Jose, CA, USA) [1]. MSC phenotype was confirmed by positivity to CD106, CD44, Sca-1 and MHC-I and negativity to CD45, CD11b and MHC-II.

### BV2 microglia culture

BV2 cells were a generous gift from Dr Thameem Dheen of the National University of Singapore. BV2 is a murine microglial cell line immortalised with v-raf/v-myc genes carrying retrovirus J2 [33]. Cells were cultured in DMEM with 5% heat-inactivated foetal bovine serum, 100 U/ml penicillin, 100 μg/ml streptomycin, 10 g/ml gentamicin (all Invitrogen), 1 × non-essential amino acids and 6.25 μg/ml insulin (Sigma-Aldrich, St. Louis, MO, USA). Cells were either sub-cultured or used for downstream assays before reaching 90% confluency by harvesting with 0.25% trypsin containing 1 mM EDTA for 5 minutes at 37°C.

### MSC/BV2 co-cultures

BV2 and MSC were seeded simultaneously at a ratio of 1:0.2 and incubated overnight at 37°C in a 5% CO_2_ incubator to allow cells to adhere. Co-cultures were then stimulated with 1 μg/ml lipopolysaccharide (LPS; *E. coli* serotype O26:B6; Sigma Cat. No. L2762). This culture set-up will be described as ‘activated co-cultures’ hereafter. The time point of LPS addition was considered as 0 hour for all experiments. Cell culture inserts with a 1 μm polyethylene terephthalate membrane pore size (Falcon, BD Biosciences, Erembodegem, Belgium) were used for transwell experiment set-up.

### ^3^H-TdR incorporation assay

BV2 cell proliferation was determined by assessing tritiated thymidine (^3^H-TdR; Perkin Elmer, Boston, USA) incorporation. In 96-well plates, 1 × 10^3^ MSC were seeded in triplicate and allowed to adhere overnight. The following day, MSC were treated with 10 μg/ml mitomycin-C (Sigma) for 2 hours to halt their proliferation. Plates were washed thoroughly with DMEM to remove any traces of the mitotic inhibitor and BV2 cells were then seeded at 5 × 10^3^ cells/well. Co-cultures were activated with 1 μg/ml LPS for 48 hours and ^3^H-TdR (0.037 MBq/well (0.5 μCi/well)) was added to wells at the final 6 hours of incubation. Plates were exposed to a freeze/thaw cycle at -20°C to ease cell harvesting. Cells were harvested onto a filter mat by using an automated cell harvester (Harvester Mach III M, TOMTEC, CT, USA Thymidine incorporation was measured by liquid scintillation spectroscopy on a beta counter (MicroBetaTriLux, Perkin Elmer Boston, USA) after the addition of scintillation fluid (OptiPhaseSuperMix Cocktail; Perkin Elmer Boston, USA) and readouts were in counts per minute (cpm).

### Griess assay

Nitric oxide (NO) was detected in the supernatant of cultures using the Griess assay. For this, 50 μl culture supernatant from each sample was transferred to a 96-well plate in triplicate and an equal volume of Griess reagent added (1% sulphanilamide/0.1% N-1-napthylethylenediamine dihydrochloride/2.5% phosphoric acid; all from Sigma). Absorbance was read at 530 nm (MRX II microplate reader, Dynex, VA, USA) after 10 minutes incubation. Nitrite concentration was calculated with reference to a standard curve of freshly prepared sodium nitrite (0 to 100 μM). The results are displayed as concentration of NO_2_^-^ in μM.

### Apoptosis assay

Apoptosis of cells in co-culture was determined by flow cytometry after double staining with FITC-Annexin-V and propidium iodide (PI). BV2 cells and MSC were co-cultured overnight at a 1:0.2 ratio, stimulated with 1 μg/ml LPS the following day, and left in culture for 48 hours. Cells were then harvested using 0.25% trypsin-EDTA. Cells were washed twice in ice-cold PBS and suspended in 100 μl of 1X binding buffer at a concentration of 1 × 10^6^ cells/ml. Cells were stained for CD45 by incubating with 0.5 μl antibody (Rat anti-mouse CD45, BioLegend®, San Diego, CA, USA ) at 4°C for 15 minutes followed by 15 minutes incubation with secondary antibody (DyLight™ 649 Goat anti-rat IgG, BioLegend**®**). CD45 staining was performed to distinguish BV2 microglia from the MSC population during flow cytometry analysis. Five microlitres each of FITC-conjugated Annexin-V and PI was added to each tube and incubated for 15 minutes at room temperature. Four hundred microlitres of 1X binding buffer was added to each tube before acquisition and analysis using a flow cytometer.

### Cell cycle analysis

Distribution of cells through the three distinct phases of cell cycle (G0/G1, S and G2/M phase) was analysed using PI staining. Co-cultures were harvested using 0.25% trypsin-EDTA and washed once in PBS by centrifugation at 1,000 rpm for 7 minutes. Cells were then suspended in 1 ml of ice-cold PBS at a density of 0.5 × 10^6^ cells per tube. The cell suspension was subsequently added drop wise to 3 ml ice-cold 95% ethanol for fixation. The tubes were incubated at -20°C for at least 2 hours. The tubes were removed from -20°C and spun at 1,200 rpm for 10 minutes and washed once using ice-cold PBS to remove traces of ethanol. The cells were then suspended in 500 μl of 50 μg/ml PI staining solution and incubated at 37°C for 15 minutes. Stained cells were washed once with PBS to remove excess PI and suspended in 400 μl PBS. The cell cycle data for individual samples were acquired using the BD LSR Fortessa™ flow cytometer equipped with BD FACSDiva™ software (BD) and analysed using ModFit LT™ software (Verity Software House, ME, USA).

### Cytokine bead array/protein array for TNF-α and IL-6 detection

Co-culture supernatants were assayed at 24 hours using a custom RayBio® mouse cytokine array kit (RayBiotech, Inc., GA, USA ), according to manufacturer’s instructions. The results are expressed as relative protein levels compared with LPS-stimulated BV2 cells. Absolute quantity of IL-6 and TNF-α in culture supernatants were then determined at 24 hours by using a multiplex bead array kit (BD Cytometric Bead Array mouse inflammation kit; BD Biosciences, San Jose, CA, USA), according to the manufacturer’s instructions. Samples were assayed on a FACS Fortessa flow cytometer (BD Biosciences) and analysed with FCAP array software (BD Biosciences). Concentration of cytokines in samples was calculated using individual standard curves and expressed as pg/ml.

### Cytokine blocking studies

Specific blocking antibodies to TNF-α and IL-6 were used to elucidate the functional importance of modulation of these cytokines in co-culture. Blocking antibodies against TNF-α (XT3.11), IL-6 (MP5-20 F3) and isotype (Rat IgG1) control (all from Bio X Cell, NH, USA) were reconstituted in 1X PBS to 1.0 mg/ml. The antibodies were then serially diluted in culture media to obtain working stock concentrations of 20.0, 2.0 and 0.2 μg/ml. Cells were plated either in 12-well plates (for Griess assays) or in 96-well plates (for proliferation assays) as described earlier. Cells were stimulated with 1 μg/ml LPS after overnight incubation. Equal volumes of diluted antibody were added to cultures to obtain a final concentration of 10.0, 1.0 and 0.1 μg/ml. Culture supernatants were collected at 24 and 48 hours and assayed for NO production using Griess assay. Proliferation was analysed at 48 hours by pulsing the cultures with ^3^H-TdR for the final 6 hours of incubation.

### Statistical analysis

Mean values and standard deviations (SD) were calculated from three independent experiments and significance was assessed using one-way analysis of variance followed by the Tukey *post hoc* test or student’s *t* test using GraphPad Prism version 6 (GraphPad software, San Diego, CA, USA).

## Results

### Mesenchymal stem cells inhibit BV2 microglia proliferation independent of nitric oxide

**Figure 1:**
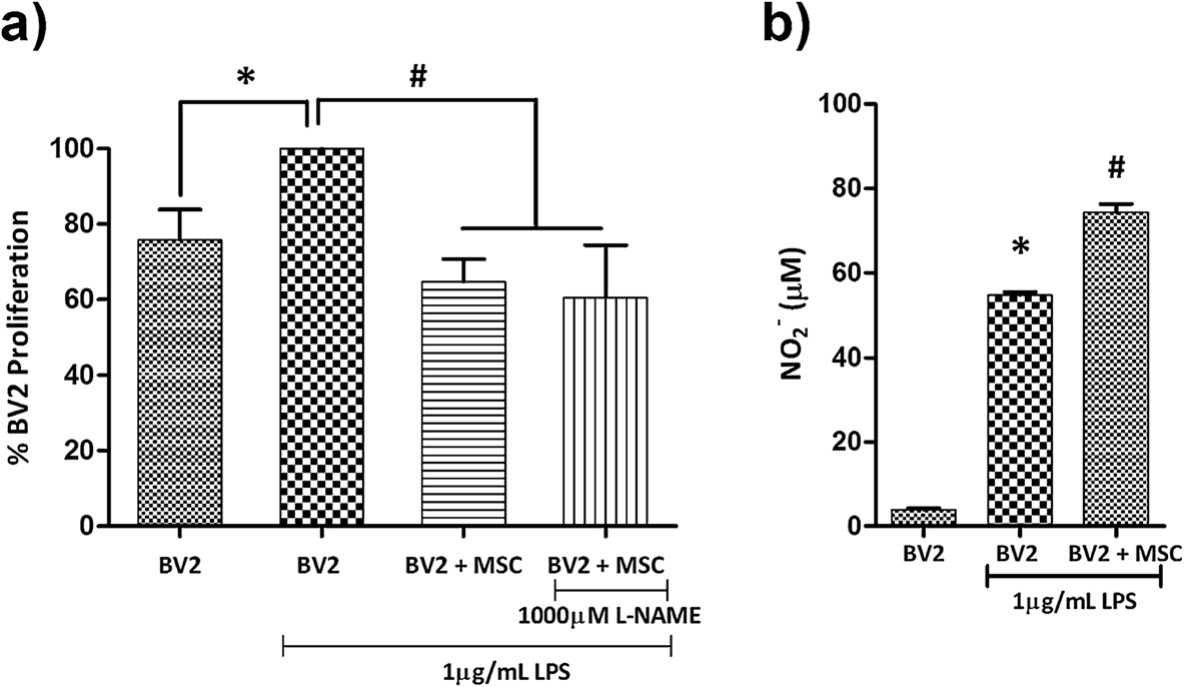
**Mesenchymal stem cells inhibits BV2 microglia proliferation independent of nitric oxide. (a)** BV2 cells and Mesenchymal stem cells (MSC) were seeded at a 1:0.2 ratio in a 96-well plate with 1 μg/ml lipopolysaccharide (LPS) and 1000 μM N-nitro-L-arginine methyl ester (L-NAME) for 48 hours, and BV2 proliferation was determined with a tritiated thymidine (^3^H-TdR) incorporation assay. Values are expressed as mean ± SD of percentage BV2 proliferation from three independent experiments. **(b)** NO_2_^-^ concentration in culture supernatant was determined using the Griess assay. BV2 cells and MSC were cultured in 12-well plates at a 0.2 ratio (BV2: MSC) and stimulated with 1 μg/ml LPS, and NO_2_^-^ was assayed at 48 hours. Values are expressed as mean ± standard deviation of NO_2_^-^ in μM from three independent experiments. *P < 0.01, versus BV2 cells; #*P* < 0.01, versus BV2 + LPS.

An *in vitro* model of microglia proliferation was established by examining BV2 microglia proliferation in response to LPS stimulation at 6, 24 and 48 hours. The proliferation rate of BV2 microglia appeared unaffected by LPS at 6 and 24 hours (Additional file 1: Figure S1a,b). At 48 hours, LPS-stimulated microglia displayed an approximate 30% increase in proliferation (Figure 1a, *P* < 0.01; Additional file 1: Figure S1c). This increase in microglia proliferation was used as a model to test the anti-proliferative effect of MSC.

To study the mechanisms through which MSC exert their anti-proliferative effect on microglia, the BV2:MSC ratio of 0.2 was selected based on previous reports from our laboratory [28,34] (see Additional file 2: Figure S2 for images of BV2/MSC co-culture). Co-culturing with MSC at a 0.2 ratio decreased LPS-induced proliferation of BV2 microglia by 28.77 ± 7.90% at 48 hours (Figure 1a, *P* < 0.01), similar to proliferation rates of unstimulated BV2 microglia. MSC were mitotically arrested by mitomycin-C (10 μg/ml) treatment (Additional file 1: Figure S1d,e) to facilitate accurate measurement of microglia proliferation in co-culture.

A previous report from our laboratory demonstrated that MSC generate NO upon exposure to soluble factors from microglia, while BV2/MSC co-culture induces a surge in NO levels beyond that of LPS-stimulated microglia [34]. When tested in this study, co-culture of BV2:MSC at a 1:0.2 ratio induced a 25% surge in NO levels from 56.94 ± 2.65 μM to 76.59 ± 3.08 μM at 48 hours (Figure 1b; *P* < 0.01). Within an immunomodulatory paradigm, NO has been shown to play a vital role in MSC-mediated suppression of activated T cell proliferation [35,36]. These reports, with the use of MSC derived from inducible nitric oxide synthase (iNOS)^-/-^ mice, have pinpointed that NO generated by MSC in the co-culture paradigm plays a vital role in reducing T cell proliferation. We used N-nitro-L-arginine methyl ester (L-NAME), a specific inhibitor of NOS, to address the role of NO in conferring the anti-proliferative effect of MSC on BV2 microglia. We tested a series of L-NAME concentrations (50, 125, 250, 500, 750 and 1000 μM) on LPS-stimulated BV2 microglia (Additional file 3: Figure S3) and demonstrated that even reducing the NO levels in co-culture to levels comparable to unstimulated BV2 microglia (using 1000 μM L-NAME) does not alter the anti-proliferative effect conferred by MSC (Figure 1a).

### Co-culture does not induce apoptosis

**Figure 2:**
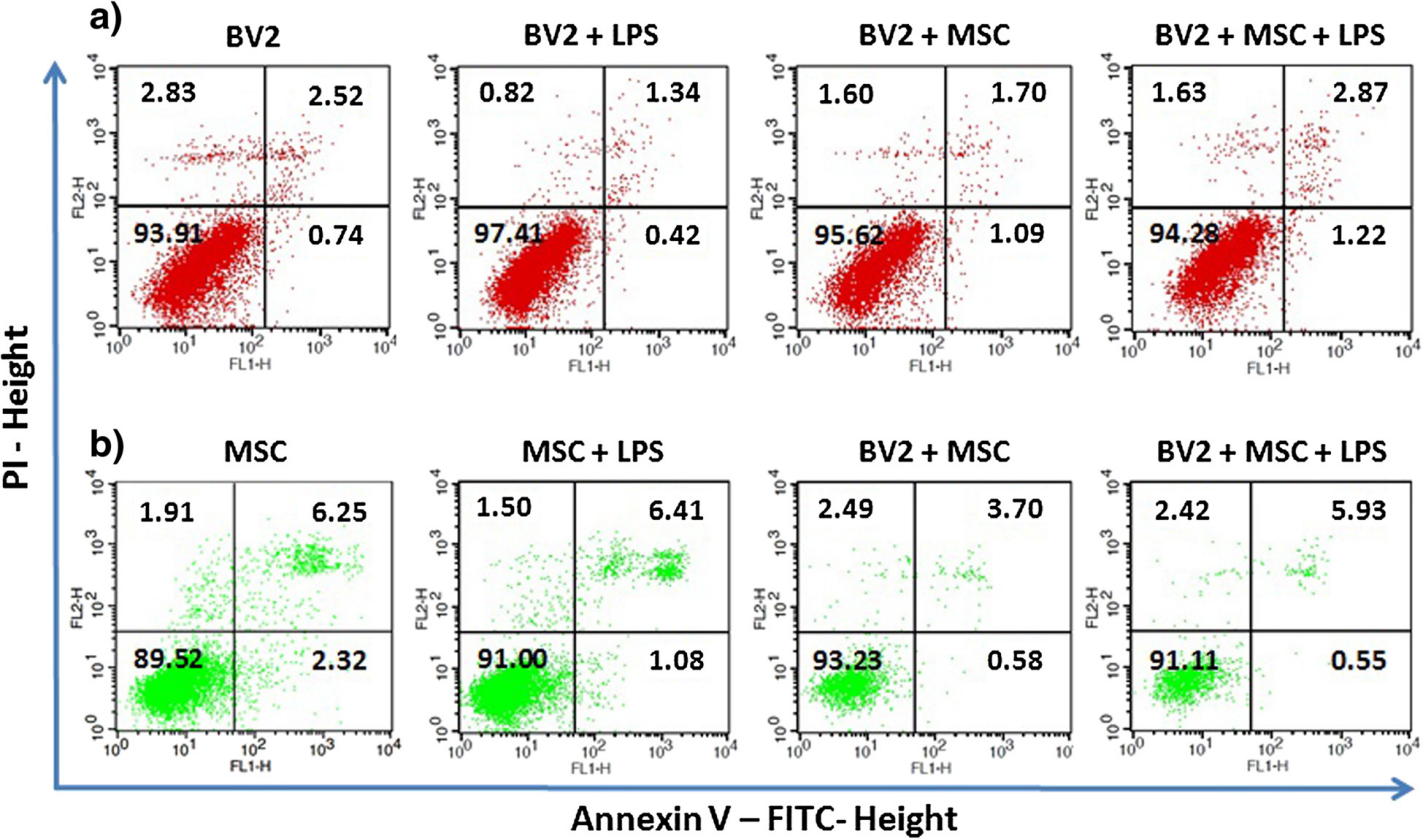
**Co-culture does not induce apoptosis in BV2 cells and mesenchymal stem cells.** Scatter plots show percentage of viable (lower left), early apoptotic (lower right), late apoptotic (upper right) and necrotic (upper left) populations in **(a)** BV2 cells and **(b)** mesenchymal stem cells (MSC) as determined by Annexin-V/propidium iodide (PI) staining 48 hours post-lipopolysaccharide (LPS) stimulation. The number within each quadrant of plot indicates percentage of cells. Results are from a representative of three independent experiments.

Data from the ^3^H-TdR assays indicate that the reduction of microglial proliferation results from a decreased number of actively proliferating cells. Such reductions could also occur due to induced death of the responder cells as reported for MSC–T cell interactions [37], apart from the inhibition of proliferation postulated here. Thus, we sought to determine whether co-culture induces cell death in microglia. When BV2 cells were co-cultured with MSC, the proportion of apoptotic cells as examined by Annexin V and PI staining (Figure 2a) of the CD45^+^ population (Additional file 4: Figure S4a,c) was not altered. The average percentage viability (Annexin-V^-^/PI^-^) of BV2 cells remained higher than 94.0 ± 1.7% (Additional file 5: Table S1) across all samples tested, ruling out the process of apoptosis as a compounding factor in inhibition of BV2 proliferation by co-culture. Similarly, no significant cell death was noted for MSC (CD45^-^ population; Additional file 4: Figure S4b,c) in co-culture (Figure 2b and Additional file 5: Table S1).

### Mesenchymal stem cells inhibit BV2 microglia proliferation through contact-dependent cell cycle modulation

**Figure 3:**
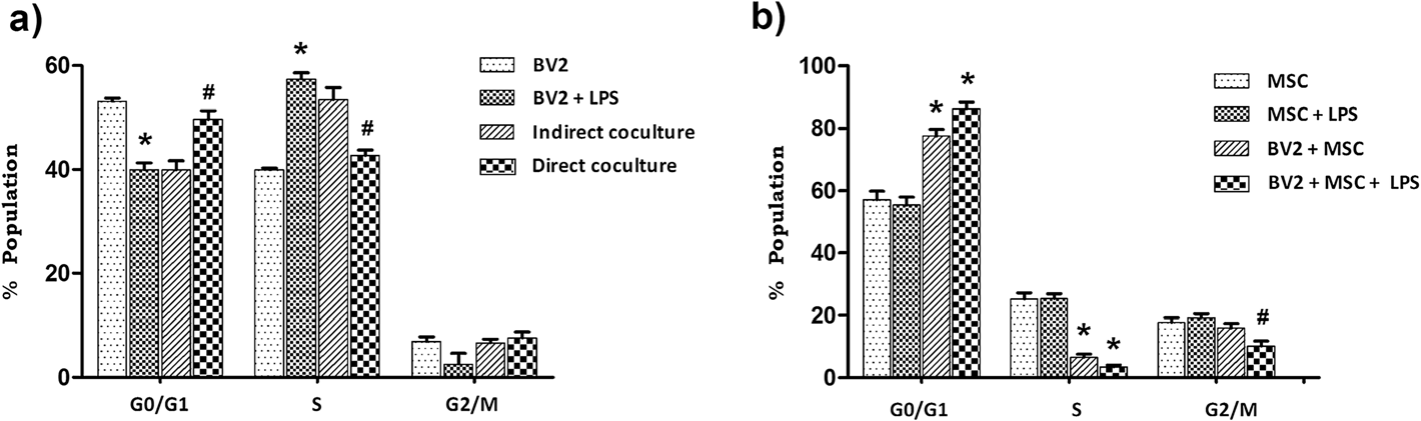
**Mesenchymal stem cells inhibit microglia proliferation through contact-dependent cell cycle modulation.** BV2 and Mesenchymal stem cells (MSC) were cultured at a 1:0.2 ratio and stimulated with 1 μg/ml lipopolysaccharide (LPS) after overnight incubation. Cells were harvested 48 hours post-LPS stimulation and cell cycle was analysed by propidium iodide staining. Separation of BV2 and MSC populations on flow cytometry was enabled by CD45 staining (see Additional file 3: Figure S3). Histograms show distribution of **(a)** BV2 cells and **(b)** MSC from direct co-culture across G0/G1, S and G2/M phases of cell cycle at 48 hours after LPS stimulation and/or co-culture. Values are expressed as mean ± standard deviation of percentage of cells from three independent experiments. **(a)** *P < 0.01, versus BV2 cells; #*P* < 0.01, versus BV2 + LPS. **(b)** *P < 0.001, versus MSC; #*P* < 0.001, versus MSC + LPS.

MSC in co-culture are shown to induce cell cycle arrest of target cells (splenocytes, dendritic cells and in tumour cells) at G0/G1 phase [12,14,38]. Experiments were performed in order to test whether MSC induce similar effects on microglia. At 48 hours, LPS stimulation induced a surge of BV2 microglia in the S phase of the cell cycle from 40.0 ± 0.4% to 57.4 ± 1.2% (*P* < 0.01) and direct co-culture with MSC reduced the percentage of BV2 microglia in S phase to 42.7 ± 1.1 (*P* < 0.01), levels comparable to resting BV2 cultures (Figure 3a). Experiments were also performed with MSC separated from BV2 cells in culture wells by transwell inserts of 1 μm pore size to determine the importance of cell-to-cell contact in cell cycle modulation. The transwell system physically separates BV2 microglia and MSC but permits the transfer of soluble factors and thus enables the demarcation of the role of soluble factors and cell-to-cell contact. MSC in transwell (in the absence of cell-to-cell contact) did not induce a significant change in the percentage population of BV2 cells in S phase (53.4 ± 2.4) compared with LPS-stimulated BV2 cells, revealing that cell-to-cell contact is critical for MSC-mediated modulation of BV2 microglia cell cycling.

In order to test whether the slowdown of LPS-stimulated BV2 proliferation in co-culture is indeed an arrest, their cell cycle was further analysed at 24, 48, 72 and 96 hours (Table 1) in separate experiments. MSC did not induce cell cycle arrest in BV2 cells at any of the time points tested but continued to restore the cell cycle of LPS-stimulated BV2 microglia to that of resting BV2 cells (BV2 microglia cells alone).

**Table 1:** **Co-culture with Mesenchymal stem cells continues to modulate BV2 cell cycle until 96 hours but does not induce a cell cycle arrest**

**Cell cycle distribution (BV2 Microglia: MSC direct coculture)**									
	**BV2**			**BV2 + LPS**			**BV2 + MSC + LPS**		
	**G0/G1**	**S**	**G2/M**	**G0/G1**	**S**	**G2/M**	**G0/G1**	**S**	**G2/M**
24 hrs	42.8 ± 1.5	49.3 ± 3.7	7.0 ± 2.5	34.2 ± 0.6^*^	62.0 ± 3.8^#^	3.8 ± 3.3	44.3 ± 1.8^†^	50.1 ± 1.0^‡^	5.6 ± 4.9
48 hrs	50.7 ± 1.9	44.2 ± 1.1	4.7 ± 1.2	38.2 ± 1.2^*^	59.0 ± 2.7^#^	1.8 ± 2.2	43.9 ± 1.9^†^	48.6 ± 1.2^‡^	6.3 ± 3.7
72 hrs	77.6 ± 0.9	19.3 ± 0.9	3.1 ± 0.1	48.2 ± 1.5^*^	51.6 ± 1.8^#^	0.3 ± 0.3	52.4 ± 5.6	41.3 ± 2.4^‡^	1.9 ± 3.0
96 hrs	83.8 ± 4.0	14.1 ± 3.7	2.1 ± 2.3	63.2 ± 1.3^*^	35.7 ± 21.1^#^	1.0 ± 1.1	75.6 ± 1.7^†^	18.4 ± 0.9^‡^	6.0 ± 1.1^€^

BV2 cells and Mesenchymal stem cells (MSC) were seeded at a 1:0.2 ratio in 6-well plates and left overnight prior to lipopolysaccharide (LPS) stimulation (1 μg/ml). Cell cycle analysis of BV2 cells (CD45 + ve population in co-culture) was performed using propidium iodide (PI) staining at the time points indicated. Data show mean percentage population of BV2 cells ± standard deviation from three independent experiments. *P < 0.05, ^#^ *P* < 0.05, versus respective BV2 alone controls. †*P* < 0.05, ‡*P* < 0.05, versus the corresponding phases in BV2 + LPS.

Interestingly, co-culture exerted a G0/G1 arrest in MSC cell cycle by 48 hours. Presence of microglia in the culture induced an approximate 20% increase of MSC at the G0/G1 phase (Figure 3b; *P* <0.001). The surge was more prominent when cultures were stimulated with LPS; LPS-activated microglia retained most of the MSC at G0/G1 phase (86.32 ± 2.05%; *P* < 0.001). Neither MSC cell cycle (Figure 3b) nor proliferation (Additional file 1: Figure S1f) was affected by exposure to LPS alone. Cell cycle arrest of MSC in co-culture may also be induced by contact inhibition and/or media deprivation. In order to negate these possibilities, the cell seeding density of both MSC and BV2 were reduced by 10 times in co-culture and MSC cell cycle was analysed. Even at such low seeding density, where probabilities of contact inhibition or media deprivation was minimal, MSC cell cycle was arrested as early as 24 hours when in co-culture with BV2 microglia (Additional file 6: Figure S5).

### Mesenchymal stem cells modulate the cytokine profile of co-cultures

**Figure 4:**
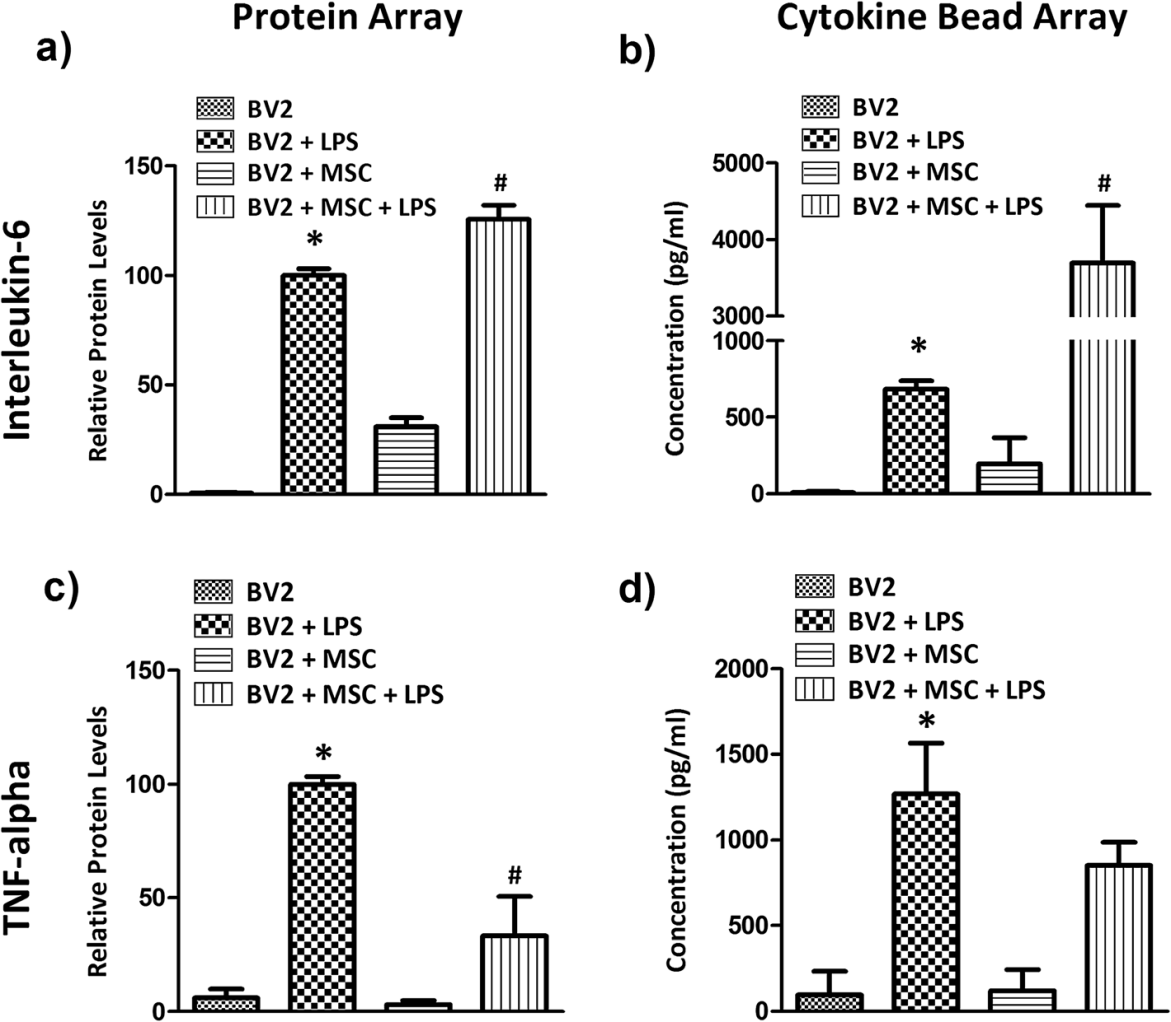
**Mesenchymal stem cells modulate the expression of IL-6 and TNF-α in microglia co-culture.** Microglia (BV2 cells or primary microglia) and Mesenchymal stem cells (MSC) were seeded simultaneously into 24-well plates at a 1:0.2 ratio. Cells were left overnight in culture and stimulated with 1 μg/ml lipopolysaccharide (LPS). Culture supernatants were collected 24 hours later and expression of **(a,b)** interleukin (IL)-6 and **(c,d)** tumour necrosis factor (TNF)-α were analysed using the RayBio antibody array (a,c) or BD Cytokine Bead Array (b,d). Protein array results are expressed as mean relative protein level ± standard deviation from two independent experiments and cytokine bead array results are expressed in mean concentration (pg/ml) ± standard deviation from three independent experiments. *P < 0.001, versus BV2 cells; #*P* < 0.001, versus BV2 + LPS.

Culture supernatants were examined using a glass slide-based protein array to identify soluble proteins involved in MSC-mediated immunomodulation of microglia. From this array it was observed that IL-6 and TNF-α were significantly modulated by co-culture. BV2 microglia in culture produced negligible amounts of IL-6 and TNF-α (Figure 4a,c). LPS stimulation increased the expression of both cytokines at 24 hours. Co-culture with MSC in the presence of LPS further induced a 25.5 ± 6.5% surge in IL-6 (Figure 4a, *P* < 0.001), whilst TNF-α levels were reduced to 33.2 ± 17.3% (Figure 4c, *P* < 0.001). These cytokines were then quantified using a cytometric bead array. Consistent with the results from protein array, IL-6 production was negligible in BV2 cultures (~9 pg/ml; Figure 4b). LPS stimulation increased IL-6 levels to 683.1 ± 55.4 pg/ml by 24 hours and the presence of MSC increased IL-6 levels to 3,693.8 ± 751.1 pg/ml in LPS-treated BV2 microglia cultures (Figure 4b). Conversely the presence of MSC reduced TNF-α levels to 852.1 ± 134.9 pg/ml compared with 1,271.2 ± 294.2 pg/ml (Figure 4d) in LPS-stimulated BV2 cells alone.

### Mesenchymal stem cell-mediated inhibition of microglial proliferation is independent of IL-6

**Figure 5:**
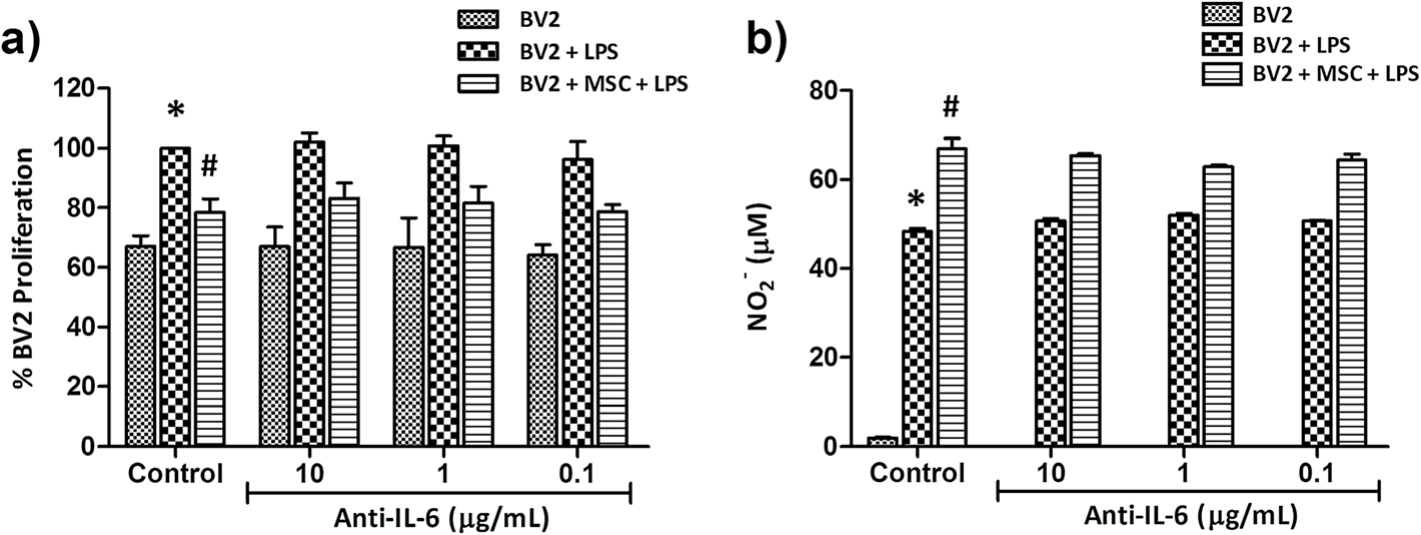
**IL-6 does not play a role in mesenchymal stem cell (MSC)-mediated modulation of nitric oxide and proliferation in BV2:MSC co-cultures.** BV2 cells and MSC were seeded at a 1:0.2 ratio into 12-well plates (Griess assay) or 96-well plates (proliferation assay). After overnight incubation, 1 μg/ml lipopolysaccharide (LPS) and interleukin (IL)-6 blocking antibodies at concentrations indicated below the graph were added to co-cultures and **(a)** proliferation and **(b)** NO_2_^-^ concentration were analysed at 48 hours. Values are expressed as mean ± standard deviation from three independent experiments. Controls are respective cultures without blocking antibodies. *P < 0.05, versus control BV2 cells; #*P* < 0.05, versus control BV2 + LPS.

LPS stimulation induced proliferation of BV2 microglia by 32.24 ± 4.5% and co-culture of BV2 microglia and MSC at the 0.2 ratio reduced LPS-stimulated proliferation of BV2 microglia by 21.50 ± 3.32% (Figure 5a, *P* < 0.05). In order to identify whether the surge in IL-6 in BV2/MSC co-culture is responsible for the anti-proliferative effect of MSC, IL-6 from cultures was neutralised using anti-mouse IL-6 blocking antibody. An isotype IgG was used as a control to negate any non-specific effects. Addition of isotype IgG did not induce any changes (data not shown). Neutralising IL-6 from cultures did not influence the proliferation of LPS-stimulated microglia nor the anti-proliferative effect of MSC (Figure 5a). It was also demonstrated that IL-6 did not influence the NO production within the MSC-mediated immunomodulatory paradigm (Figure 5b).

### Mesenchymal stem cells inhibit microglial proliferation by reducing TNF-α

**Figure 6:**
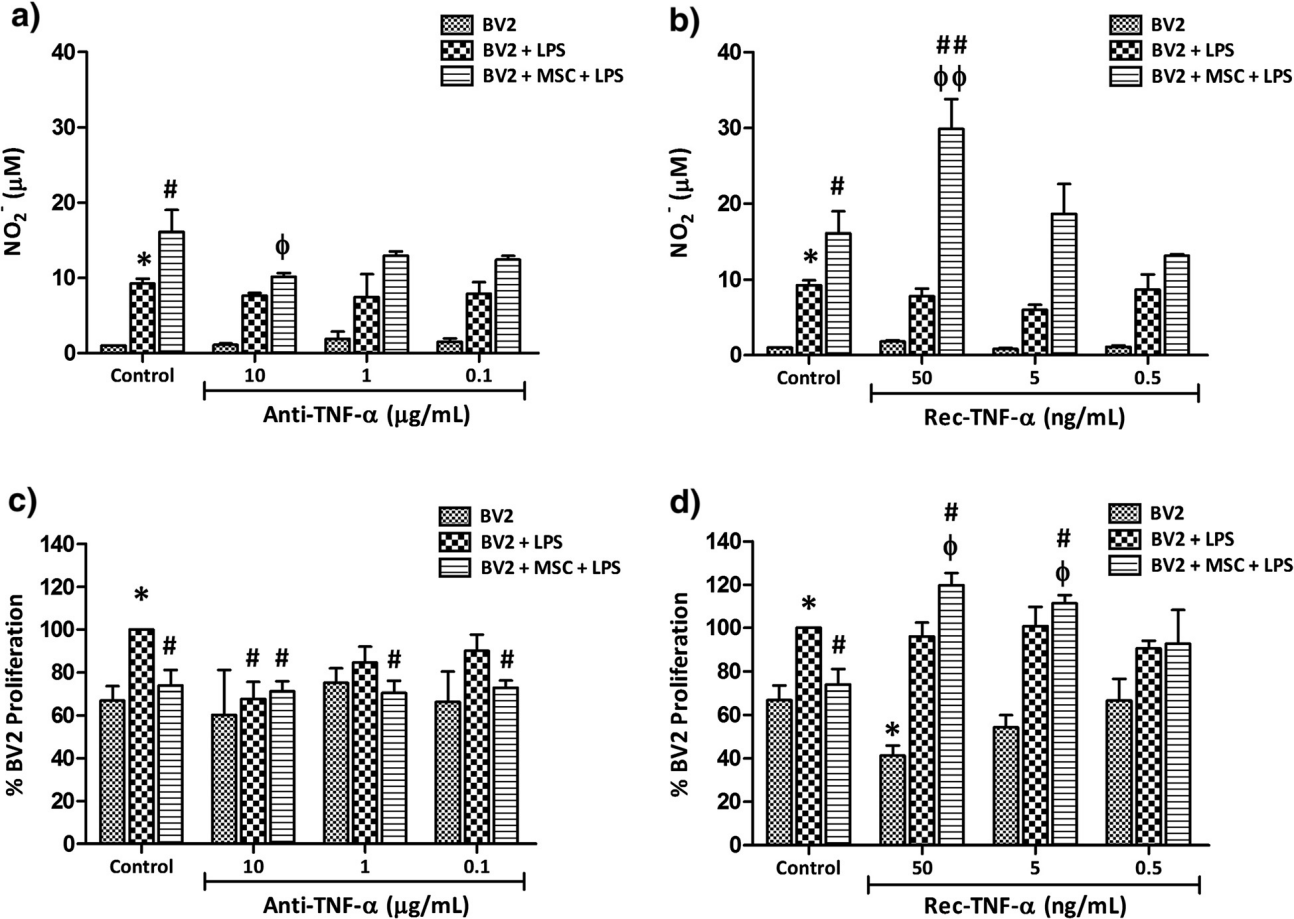
**Nitric oxide surge in co-culture and reduction in microglial proliferation are mediated by TNF-α modulation.** BV2 cells and mesenchymal stem cells (MSC) were seeded at a 0.2 ratio into 96 well plates. After overnight incubation, 1 μg/ml lipopolysaccharide (LPS) and **(a,c)** tumour necrosis factor (TNF)-α blocking antibodies or **(b,d)** recombinant TNF-α were added at concentrations indicated below the graph, and NO_2_^-^ concentration (a,b) and proliferation (c,d) was analysed at 48 hours. Values are expressed as mean ± standard deviation from three independent experiments. Controls are culture wells without blocking antibodies. *P < 0.05, versus control BV2 cells; #*P* < 0.05, ##*P* < 0.001, versus control BV2 + LPS; ф*P* < 0.05, фф*P* < 0.001, versus control co-culture + LPS.

We sought to determine the role of TNF-α in MSC-mediated immunomodulation of microglial responses and first addressed whether application of blocking antibodies could affect the NO profile. Similar to that demonstrated previously, co-culture induced a surge in LPS-induced NO production (Figure 6a). Neutralising TNF-α did not affect NO levels in LPS-stimulated BV2 cultures, but 10 μg/ml TNF-α blocking antibody significantly reduced NO levels in co-culture (Figure 6a, *P* < 0.05). Next, we applied recombinant TNF-α to co-cultures and its effect on NO expression was studied. TNF-α levels detected in LPS-stimulated BV2 cultures by the cytokine bead array were approximately 1.5 ng/ml. Addition of recombinant TNF-α at low concentrations (0.5 and 5.0 ng/ml) did not affect the NO profile of any of the culture conditions tested (Figure 6b). However, at 50 ng/ml, TNF-α induced NO levels in co-culture by approximately two-fold (Figure 6b, *P* < 0.001 compared to activated co-culture without recombinant TNF-α).

Next we sought to address the effect of TNF-α deprivation on microglial proliferation. LPS stimulation induced the proliferation of BV2 microglia by approximately 30% and in co-culture MSC reduced the proliferation of LPS-stimulated BV2 microglia by 26.13 ± 7.24% (Figure 6c, *P* < 0.05). Neutralising TNF-α in LPS-stimulated BV2 microglia cultures (without MSC) reduced their proliferation in a dose-dependent manner; at 10 μg/ml, proliferation was reduced to levels similar to unstimulated BV2 microglia. As MSC reduced TNF-α levels (Figure 4c,d) in LPS-stimulated BV2 microglia cultures, we presume that a reduction of TNF-α by MSC may be the mechanism through which MSC exert their anti-proliferative effect on microglia. Further reduction in TNF-α levels did not influence the anti-proliferative effect of MSC; percentage proliferation of BV2 remained between 70.57 ± 5.6% and 72.70 ± 3.5% for all three antibody concentrations tested (Figure 6c). Addition of recombinant TNF-α to co-cultures abolished the anti-proliferative effect of MSC at concentrations as low as 0.5 ng/ml, with higher concentrations inducing a dose-dependent increase in microglial proliferation (Figure 6d). At 50 ng/ml TNF-α, microglia proliferation was induced in co-culture to 119.75 ± 5.56% (Figure 6d, *P* < 0.05) compared to 73.87 ± 7.24% in the control co-culture.

On the contrary, TNF-α exerted an anti-proliferative effect on resting BV2 microglia (Figure 6d). Addition of TNF-α to unstimulated BV2 culture at 50 ng/ml reduced their proliferation to 41.26 ± 4.62% (Figure 6d, *P* < 0.05) compared to 66.94 ± 6.58% in the control, whereas addition of TNF-α did not alter the proliferation of LPS-induced BV2 microglia.

## Discussion

Here, for the first time, we demonstrate the role of TNF-α in MSC-mediated immunomodulation of microglia. Treatment of BV2 microglia cultures with TNF-α blocking antibodies reduced LPS-induced proliferation while addition of recombinant TNF-α to co-cultures abolished the anti-proliferative effect of MSC on microglia. Microgliosis is a common response towards injury in the CNS, including Alzheimer’s disease [39], Parkinson’s disease [40], multiple sclerosis [41] and stroke [42], and the findings here may prove to be beneficial for these conditions.

In addition, the present study negated the role of apoptosis and soluble factors such as NO and IL-6 in conferring this effect. We also provide the first evidence that MSC confer the inhibitory effect on microglia proliferation through modulation of microglia cell cycle and MSC themselves undergo a G0/G1 arrest while exerting this effect.

Following co-culture with MSC, microglia proliferation in response to LPS stimulation was dampened and NO levels were increased. Surge in NO levels in MSC co-cultures have been previously identified as the key mechanism conferring anti-proliferative effects on T cells [35,36]. To examine similar prospects, NO production in co-culture was abolished using a specific iNOS inhibitor - L-NAME - and microglia proliferation was analysed. Converse to the reports in MSC/T cell co-cultures, abolishing NO did not restore microglia proliferation in our study. This is contradictory to a recent report which suggested the role of NO in MSC-mediated inhibition of microglia proliferation [43]. In the mentioned report, conditioned media from MSC cultures treated with an iNOS inhibitor was used to study the anti-proliferative effect, and thus the role of microglial cues that may be required by MSC to exert such effects was not negated. Our laboratory has previously reported that MSC requires microglial cue to induce a NO surge [34]. The current approach to eliminate NO directly from co-culture by addition of L-NAME efficiently negates such doubts and rules out the role of NO in inhibition of microglial proliferation by MSC.

The inhibition of microglial proliferation reported here could also be contributed by cell death. MSC are shown to induce indoleamine 2,3-dioxygenase-dependent apoptosis of activated T cells [37]. Through examination using Annexin-V/PI staining, we demonstrate that co-culture with MSC does not induce apoptosis in microglia. Such differences in interaction of MSC with T cells and microglia emphasises diversity in the modulatory functions that MSC exert on different target cell types.

Next, we examined whether co-culture induces cell cycle modulation in microglia. Studies have shown MSC induce cell cycle arrest in dendritic cells [14] and tumour cells [38]. Here, for the first time, we provide evidence that MSC exert their anti-proliferative effect through modulation of microglia cell cycle. Co-culture with MSC restored the percentage of BV2 cells in the different phases of the cell cycle to levels comparable to resting microglia. Interestingly, this modulation was dependent on cell-to-cell contact. From a therapeutic perspective, we presume that suppression of the proliferative response of microglia through cell cycle modulation is beneficial, as inducing apoptosis or a permanent cell cycle arrest may perturb the volume of glia and hence affect homeostasis of the brain. Interestingly, MSC entered a G0/G1-phase cell cycle arrest while exerting the anti-proliferative effect suggesting that the inhibitory effect of MSC on microglia activation (at least in terms of microglia proliferation) is not dependent on a need for a proliferative response from MSC. The ability of MSC to modulate the proliferative response of microglia whilst simultaneously entering a cell cycle arrest is presumably a beneficial one. Tumourigenic transformation of MSC has been implicated in several experimental transplantations including osteogenic sarcomas [44], myocardial infarction and diabetic neuropathy [45]. Therefore, careful evaluation and understanding of proliferative potential of MSC within the inflamed milieu is necessary for the success of clinical interventions.

We have also demonstrated here that MSC modulate the expression of TNF-α and IL-6. Co-culture with MSC significantly reduced the TNF-α which was upregulated upon LPS stimulation whereas co-culture induced a surge in IL-6. Similar modulation of cytokines was also reported previously by us [34] and others [29,43]. The impact of differential modulation of IL-6 and TNF-α was further deduced using blocking antibodies and recombinant TNF-α. Through addition of blocking antibodies, we have identified that the IL-6 surge in co-culture does not influence the anti-proliferative effect of MSC or NO modulation. Distinct from IL-6, TNF-α seems to play a context-dependent role on modulation of NO and microglial proliferation in co-culture. Reducing TNF-α level in culture using blocking antibodies did not influence the NO profile of activated microglia. However, addition of recombinant TNF-α induced a dose-dependent NO surge in co-cultures. It has been previously described by our group that soluble factors from activated co-culture induce NO production in MSC and these soluble factors include TNF-α [34]. It is possible that, in co-culture, the additional TNF-α added to the co-culture acts in tangent with microglial signals to induce a surge in NO, or TNF-α itself acts as the signal to induce NO production by MSC. MSC are known to respond to TNF-α signals and elicit downstream responses including NFκB translocation which is required for iNOS transcription [46]. However, such postulations need to be further validated.

We also showed distinct responses in microglia proliferation upon altering TNF-α levels in culture. TNF-α has been previously identified as vital for beta-amyloid-induced proliferation of microglia; abolishing TNF signals through addition of anti-TNF-α antibody or a soluble TNF receptor inhibitor both prevented beta-amyloid-induced proliferation of microglia [47]. Similarly in an MPTP mouse model of Parkinson’s disease, knocking out TNF-α significantly reduced microglial activation as measured by cell number and morphology compared to wild-type controls [48], suggesting the important role played by TNF-α in microglial activation. Managing TNF-α levels in the inflammatory milieu is thus considered an efficient way to modulate inflammation [48,49]. At present, there is no evidence to suggest that modulation of TNF-α is the mechanism through which MSC confer an anti-proliferative effect on microglia. Likewise, there is no report on how MSC reduce microglial TNF-α. However, earlier reports from our laboratory [34] and the present study confirms the potential of MSC to downregulate TNF-α production in an LPS-stimulated microglia co-culture paradigm. Similarly, intravenous transplantation of MSC into rat traumatic brain injury models significantly decreased TNF-α levels in the injured cortex and decreased the number of glial cells at the site of injury [50]. Thus, we hypothesised that modulation of TNF-α by MSC may be vital in conferring their anti-proliferative effect and monitored the effects of neutralising TNF-α and adding recombinant TNF-α on proliferation of LPS-stimulated BV2 microglia in co-culture. Abolishing TNF-α from co-culture did not enhance the anti-proliferative effect of MSC, indicating that a further decrease of TNF-α from that seen in co-cultures does not have an additive effect on MSC anti-proliferation of BV2. Also, the basal proliferation of BV2 cells may not require TNF-α, as addition of anti-TNF onto unstimulated BV2 did not affect their proliferation. However, the proliferation of LPS-stimulated microglia was reduced in a dose-dependent manner upon addition of TNF-α neutralising antibodies, thus confirming the role of TNF-α in inducing proliferation of microglia. It is noteworthy that addition of recombinant TNF-α to LPS-stimulated BV2 culture did not increase their proliferation. This indicates that a critical concentration of TNF-α may be required for microglial activation above which the cells may become non-responsive. Also, other cues may be necessary for a proliferative response. Considering the requirement of cell-to-cell contact in modulating the cell cycle of activated microglia, we suggest the involvement of one or few cell surface molecules that may work in tandem with TNF-α modulation to achieve the inhibition of microglia proliferation by MSC. Recent studies have strongly suggested the role of cell surface molecules such as CD200 [51], HLA-G [52] and PD-1 [53] expressed by MSC on their immunomodulatory potential. It is possible that these cell surface ligands could be involved in the MSC-mediated modulation of BV2 microglia proliferation.

Next, in order to confirm the relation of MSC-mediated reduction of TNF-α and inhibition of microglial proliferation in co-culture, we used recombinant TNF-α. At concentrations as low as 0.5 ng/ml, TNF-α abolished the inhibition of microglia proliferation in co-culture. Moreover, higher concentrations of recombinant TNF-α induced the proliferation of microglia in co-culture to levels beyond that of LPS-stimulated culture. This confirms downregulation of TNF-α in co-culture by MSC as the key mechanism leading to the inhibition of microglial proliferation.

## Conclusions

Our results demonstrate the role of TNF-α in MSC-mediated immunomodulation of microglia. In addition, the present study negated the role of apoptosis and the soluble factors NO and IL-6 in conferring MSC-mediated inhibition of microglia proliferation. We also provide evidence that MSC confer the inhibitory effect on microglia proliferation through modulation of microglia cell cycle and MSC themselves undergo a G0/G1 arrest while exerting this effect. These findings suggest the potential use of MSC in modulating microglial responses in inflammatory conditions.

## Additional files

Additional file 1: Figure S1. Effect of LPS on BV2 and MSC cell proliferation and mitomycin-C on MSC. MSCs and BV2 microglia were co-cultured at a 1:0.2 ratio and proliferation was analysed with ^3^H-TdR incorporation at **(a)** 6 hours, **(b)** 24 hours and **(c)** 48 hours after stimulation with 1 μg/ml LPS. MSC were cultured independently at the same seeding density and treated with 10 μg/ml mit-C for 2 hours after overnight culture. **(d)** Proliferation and (e) cell cycle was analysed 48 hours post mit-C treatment with ^3^H-TdR incorporation and PI staining respectively. **(f)** MSC culture was stimulated with 1 μg/ml LPS and proliferation was analysed with ^3^H-TdR incorporation at 48 hours post-LPS stimulation. Values are expressed as mean ± SD from three independent experiments. **P* < 0.05, versus respective controls. ^3^H-TdR, tritiated thymidine; LPS, lipopolysaccharide; mit-C, mitomycin C; MSC, mesenchymal stem cells; PI, propidium iodide; SD, standard deviation. Additional file 2: Figure S2. Morphology of BV2 cells and MSC in culture. Phase contrast images showing morphology of BV2 cells and MSC seeded at a 1:0.2 ratio in a 6-well plate with 1 μg/ml LPS at 48 hours. LPS, lipopolysaccharide; MSC, mesenchymal stem cells. Additional file 3: Figure S3. L-NAME inhibits NO in a dose dependent manner. NO_2_^-^ concentration in culture supernatant was determined using the Griess assay at 24 and 48 hours. BV2 cells were stimulated with 1 μg/ml LPS in the presence of different concentrations of L-NAME as indicated below the graph and NO was assayed. Values are expressed as mean ± SD of three independent experiments. **P* < 0.01, versus BV2 cells; #*P* < 0.01, versus BV2 + LPS. LPS, lipopolysaccharide; L-NAME, N-nitro-L-arginine methyl ester; NO, nitric oxide; SD, standard deviation. Additional file 4: Figure S4. BV2 microglia and MSC show distinct CD45 expression. Scatter plots show CD45 expression of **(a)** BV2 microglia, **(b)** MSC and **(c)** co-culture after 48 hours in culture. **(d)** Distribution of viable (lower left), early apoptotic (lower right), late apoptotic (upper right) and necrotic (upper left) population in DMSO treated BV2 cells (positive control for apoptosis assay) as determined by Annexin-V/PI staining. Numbers within each quadrant of plot indicate percentage of cells. Results are from a representative of three independent experiments. MSC, mesenchymal stem cells; PI, propidium iodide. Additional file 5: Table S1. Co-culture does not induce apoptosis in BV2 cells and MSC. Table shows apoptosis assay results of BV2 microglia and MSC at 48 hours after co-culture. Results are expressed as mean ± SD from three independent experiments. BV2, BV2 microglia; LPS, lipopolysaccharide; MSC, mesenchymal stem cells; SD, standard deviation. Additional file 6: Figure S5. Microglia-induced cell cycle arrest of MSC is not because of contact inhibition or nutrient deprivation. BV2 and MSC were co-cultured at a 1:0.2 ratio at a seeding density 10-fold lower than usual and were stimulated with 1 μg/ml LPS after overnight incubation. Cells were harvested 48 hours post-LPS stimulation and cell cycle was analysed by propidium iodide staining. Separation of BV2 and MSC populations on flow cytometry was enabled by CD45 staining. LPS, lipopolysaccharide; MSC, mesenchymal stem cells.

## Abbreviations

CNS: central nervous system
DMEM: Dulbecco's modified Eagle's medium
^3^H-TdR: tritiated thymidine
Ig: immunoglobulin
IL: interleukin
iNOS: inducible nitric oxide synthase
L-NAME: N-nitro-L-arginine methyl ester
LPS: lipopolysaccharide
MSC: mesenchymal stem cells
NO: nitric oxide
PBS: phosphate-buffered saline
PI: propidium iodide
TNF: tumour necrosis factor
