# Supplementary figures and images for: Mesenchymal stem cells exert anti-proliferative effect on lipopolysaccharide-stimulated BV2 microglia by reducing tumour necrosis factor-α levels
Source: J Neuroinflammation. 2014 Sep 3;11:149. doi: 10.1186/s12974-014-0149-8 (PMC4156657; doi:10.1186/s12974-014-0149-8)

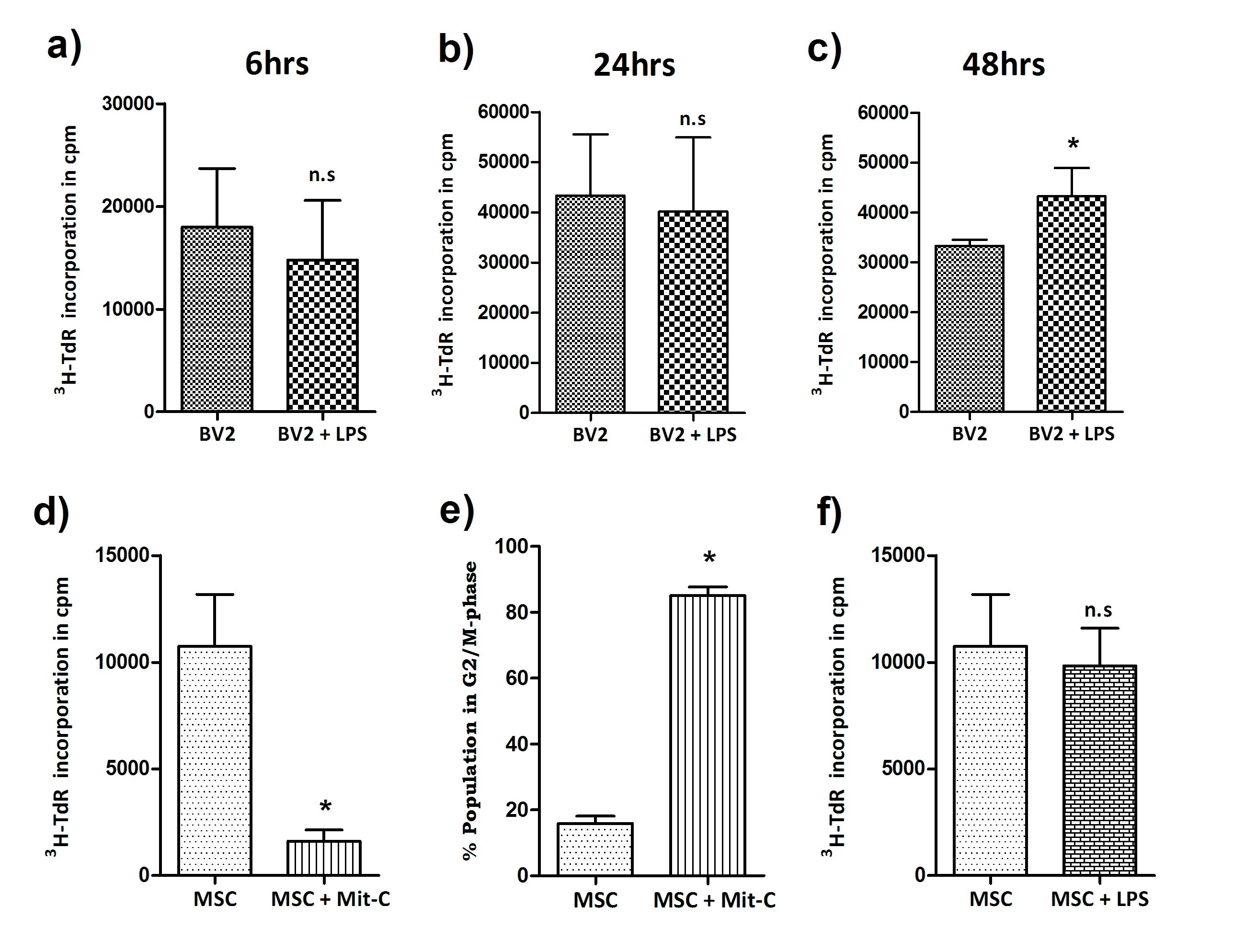

Supplement: Additional file 1: Figure S1. — Effect of LPS on BV2 and MSC cell proliferation and mitomycin-C on MSC. MSCs and BV2 microglia were co-cultured at a 1:0.2 ratio and proliferation was analysed with 3H-TdR incorporation at (a) 6 hours, (b) 24 hours and (c) 48 hours after stimulation with 1 μg/ml LPS. MSC were cultured independently at the same seeding density and treated with 10 μg/ml mit-C for 2 hours after overnight culture. (d) Proliferation and (e) cell cycle was analysed 48 hours post mit-C treatment with 3H-TdR incorporation and PI staining respectively. (f) MSC culture was stimulated with 1 μg/ml LPS and proliferation was analysed with 3H-TdR incorporation at 48 hours post-LPS stimulation. Values are expressed as mean ± SD from three independent experiments. *P < 0.05, versus respective controls. 3H-TdR, tritiated thymidine; LPS, lipopolysaccharide; mit-C, mitomycin C; MSC, mesenchymal stem cells; PI, propidium iodide; SD, standard deviation. [file 12974_2014_149_MOESM1_ESM.tiff]

## Slide 1
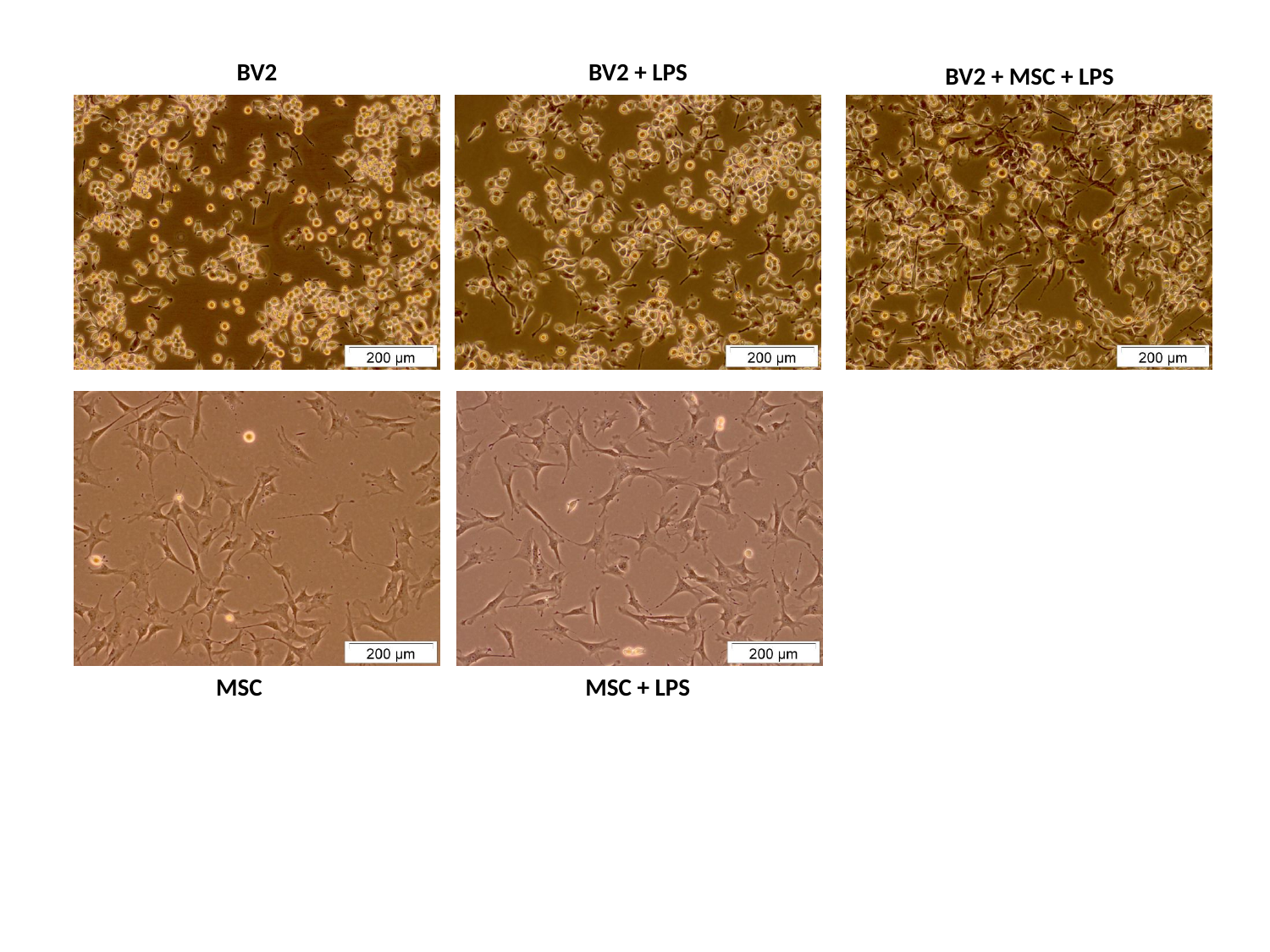

BV2 + LPS
BV2
BV2 + MSC + LPS
MSC + LPS
MSC

Supplement: Additional file 2: Figure S2. — Morphology of BV2 cells and MSC in culture. Phase contrast images showing morphology of BV2 cells and MSC seeded at a 1:0.2 ratio in a 6-well plate with 1 μg/ml LPS at 48 hours. LPS, lipopolysaccharide; MSC, mesenchymal stem cells. [file 12974_2014_149_MOESM2_ESM.pptx]

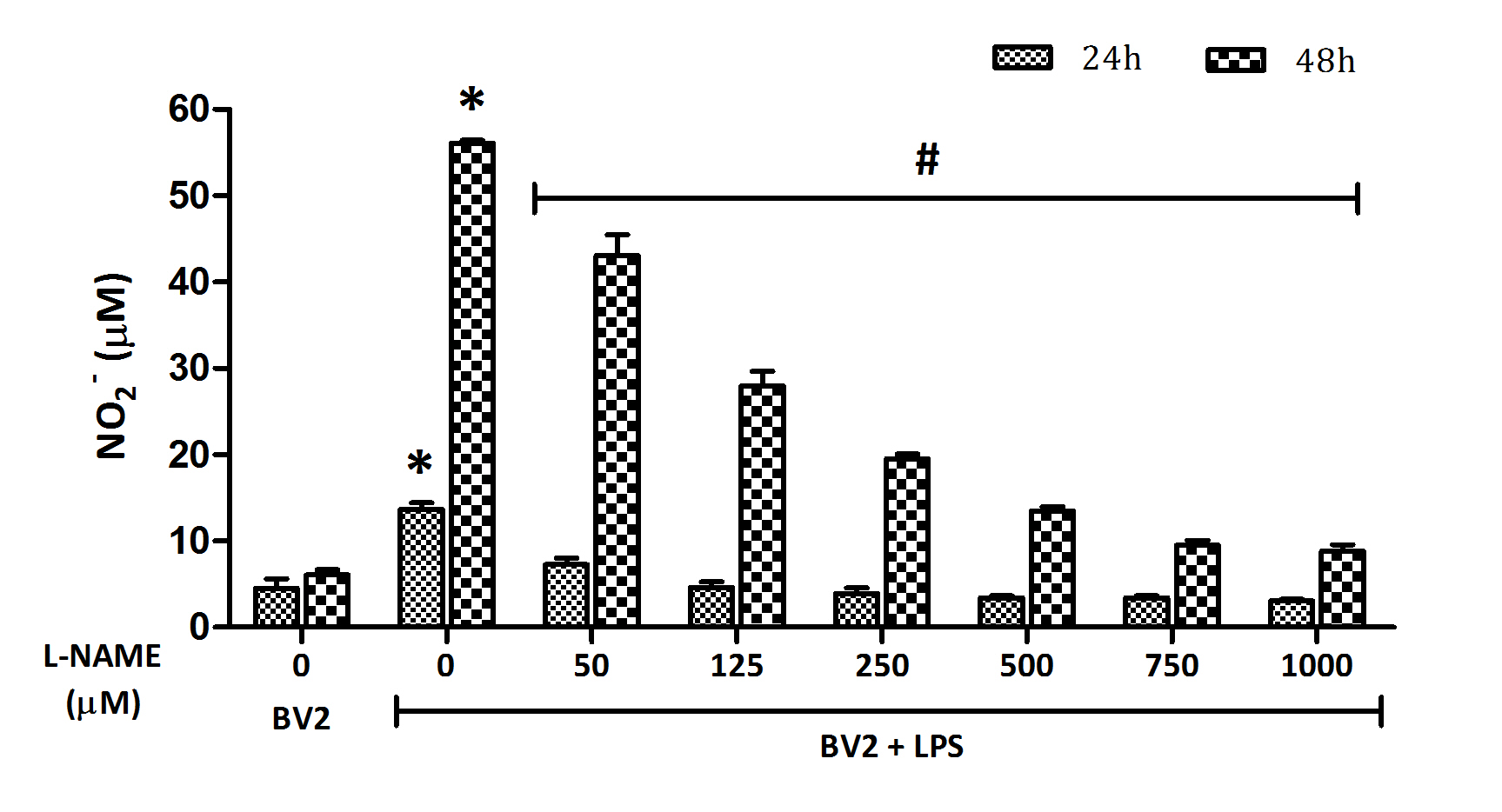

Supplement: Additional file 3: Figure S3. — L-NAME inhibits NO in a dose dependent manner. NO2 - concentration in culture supernatant was determined using the Griess assay at 24 and 48 hours. BV2 cells were stimulated with 1 μg/ml LPS in the presence of different concentrations of L-NAME as indicated below the graph and NO was assayed. Values are expressed as mean ± SD of three independent experiments. *P < 0.01, versus BV2 cells; #P < 0.01, versus BV2 + LPS. LPS, lipopolysaccharide; L-NAME, N-nitro-L-arginine methyl ester; NO, nitric oxide; SD, standard deviation. [file 12974_2014_149_MOESM3_ESM.jpeg]

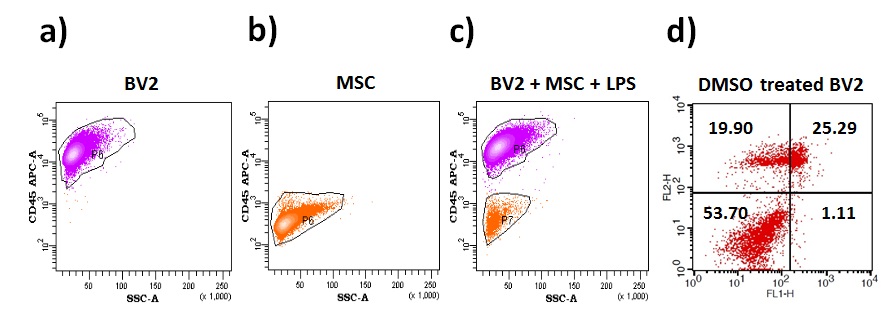

Supplement: Additional file 4: Figure S4. — BV2 microglia and MSC show distinct CD45 expression. Scatter plots show CD45 expression of (a) BV2 microglia, (b) MSC and (c) co-culture after 48 hours in culture. (d) Distribution of viable (lower left), early apoptotic (lower right), late apoptotic (upper right) and necrotic (upper left) population in DMSO treated BV2 cells (positive control for apoptosis assay) as determined by Annexin-V/PI staining. Numbers within each quadrant of plot indicate percentage of cells. Results are from a representative of three independent experiments. MSC, mesenchymal stem cells; PI, propidium iodide. [file 12974_2014_149_MOESM4_ESM.jpeg]

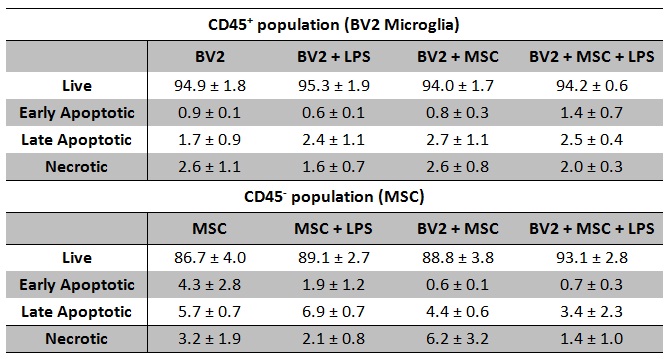

Supplement: Additional file 5: Table S1. — Co-culture does not induce apoptosis in BV2 cells and MSC. Table shows apoptosis assay results of BV2 microglia and MSC at 48 hours after co-culture. Results are expressed as mean ± SD from three independent experiments. BV2, BV2 microglia; LPS, lipopolysaccharide; MSC, mesenchymal stem cells; SD, standard deviation. [file 12974_2014_149_MOESM5_ESM.jpeg]

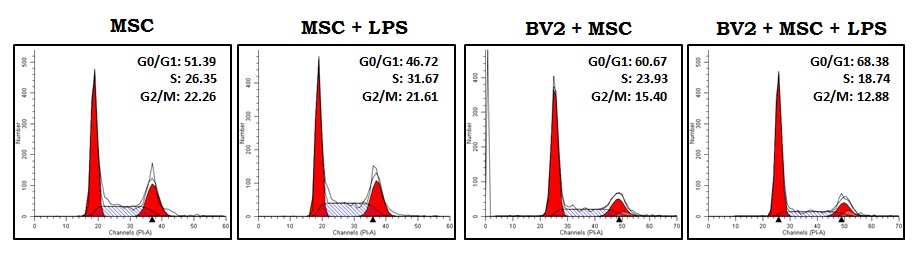

Supplement: Additional file 6: Figure S5. — Microglia-induced cell cycle arrest of MSC is not because of contact inhibition or nutrient deprivation. BV2 and MSC were co-cultured at a 1:0.2 ratio at a seeding density 10-fold lower than usual and were stimulated with 1 μg/ml LPS after overnight incubation. Cells were harvested 48 hours post-LPS stimulation and cell cycle was analysed by propidium iodide staining. Separation of BV2 and MSC populations on flow cytometry was enabled by CD45 staining. LPS, lipopolysaccharide; MSC, mesenchymal stem cells. [file 12974_2014_149_MOESM6_ESM.jpeg]
